# SHCBP1 Promotes the Proliferation of Breast Cancer Cells by Inhibiting CXCL2

**DOI:** 10.7150/jca.88072

**Published:** 2023-10-16

**Authors:** Xiaoke Yu, Guang Feng, Rui Nian, Shuai Han, Meiling Ke, Ling Wang, Wanjun Li, Shan Tian, Hongzhao Lu

**Affiliations:** 1 School of Biological Science and Engineering, Shaanxi University of Technology, Hanzhong, Shaanxi, China.; 2 Qinba State Key Laboratory of Biological Resources and Ecological Environment, Shaanxi University of Technology, Hanzhong, Shaanxi, China.; 3 Department of Biology, QinLing-Bashan Mountains Bioresources Comprehensive Development C. I. C., Shaanxi University of Technology, 723001 Hanzhong, China.; 4 Shaanxi Province Key Laboratory of Bio-resources, Shaanxi University of Technology, Hanzhong, Shaanxi, China.; 5 Affiliated 3201 Hospital of genertec Universal MedicalGroup Company Limited, Hanzhong 723000, China.

**Keywords:** Breast cancer, SHCBP1, MCF-7 cells, CXCL2, Inflammation

## Abstract

Breast cancer has the characteristics of high metastasis and recurrence and ranks first in incidence and mortality among female malignant tumors. Shc SH2-domain binding protein 1 (SHCBP1) is an important protein in intracellular signal transduction and cell division, but the role of SHCBP1 in breast cancers remains elusive. Here, we found that SHCBP1 deficiency inhibited the proliferation of breast cancer cells. Mechanistically, SHCPB1 significantly downregulates the mRNA level of CXCL2, which in turn activates the AKT and ERK signaling, while inactivates the p21 and p27 signaling. In addition, overexpression of SHCPB1 downregulates the protein levels of p21 and p27, which could be completely reversed by restoration of CXCL2 expression. Moreover, we analyzed the expression of both SHCPB1 and CXCL2, and found that SHCPB1 is highly expressed in breast cancer cells or tissues from breast cancer patients compared to normal breast cells or adjacent normal tissues, while CXCL2 is lowly expressed in breast cancer cells or tissues. Collectively, our study reveals that SHCBP1 plays an oncogenic role in breast cancer tumorigenesis partially through inhibiting the inflammatory response and ultimately activating the proliferation of breast cancers.

## Introduction

Breast cancer is a kind of malignant tumor caused by uncontrolled proliferation of breast epithelial cells and subsequent malignant lesions 1. Among the malignant tumors of women in the world, breast cancer ranks first in morbidity and mortality, and it has become the "number one killer" that endangers the physical and mental health of women around the world 2. At present, the molecular mechanism of the occurrence and development of breast cancer is more complex, and thus far, there is no definite conclusion. Studies have shown that the pathogenic factors of breast cancer mainly include genetic variation, abnormal gene expression, immune dysfunction, carcinogen exposure and emotional irritability 3-7. Because breast cancer is a heterogeneous tumor that is often accompanied by an imbalance in the expression of proto-oncogenes and tumor suppressor genes, the treatment of breast cancer is mainly surgery supplemented by radiotherapy, chemotherapy and molecular targeted therapy 8-11. Although the treatment technology of breast cancer has been improved, the clinical treatment effect of breast cancer is often poor because of its difficult to find, easy metastasis and high recurrence rate, so the overall prognosis of breast cancer patients is not particularly ideal 12. Therefore, the search for new molecular targets and biomarkers can provide a theoretical basis for early diagnosis and improvement of prognosis of patients with breast cancer.

SHCBP1 is a member of the Src homologous protein and collagen homologous protein (SHC) family. The SRC (Src homology 2 domain containing) family contains four subgene families: SHC A, SHC B, SHC C and SHC D 13. Many studies have confirmed that SHCBP1, as a proto-oncogene, is abnormally highly expressed in a variety of malignant tumors 14-15. Az et al. 16 found that SHCBP1 promotes cisplatin-induced apoptosis resistance, migration and invasion by activating the Wnt signaling pathway and then promotes the development of lung cancer. Peng et al. 17 have shown that SHCBP1 is highly expressed in synovial sarcoma tissues and cells. Overexpression of SHCBP1 can promote synovial sarcoma cell proliferation and tumorigenicity *in vitro*. In addition, the interaction between SHCBP1 and FGF13 can activate AKT/GSK3 α/β signaling pathways that promote A549 cell proliferation 18. Notably, SHCBP1 is highly expressed in breast cancer tissues and cells, but the biological function and mechanism of SHCBP1 in breast cancer cells are not clear. Whether SHCBP1 is a new oncogene in the malignant process of breast cancer still needs to be further studied.

Interestingly, the link between inflammation and cancer is bidirectional, and inflammatory cytokines can bind to specific receptors on tumor cells and promote the proliferation of tumor cells by upregulating the expression of anti-apoptosis-related genes 19. Chemokines are a class of inflammatory cytokines with chemotactic effects that can recruit immune cells to the site of the immune response and participate in immune regulation and immunopathology 20, 21. According to the special structure of amino acids and the sequence of cysteine residues (Cys) of conserved sequences, chemokines can be divided into four subgroups: C, CC, CXC and CX3C 22. CXC chemokine ligand protein 2 (CXCL2) belongs to the ELR+CXC chemokines in the CXC family, also known as macrophage inflammatory protein-2 (MIP-2) or growth regulatory protein-β (GRO-β) 23. Current studies have shown that CXCL2 is expressed in a variety of tumors, including prostate cancer, liver cancer, colorectal cancer, and lung cancer 24, 25. CXCL2 expression was higher in colorectal cancer tissues than in adjacent normal tissues and was associated with the degree of invasion and lymph node metastasis of colorectal cancer 26. In a qPCR assay of 23 genes in non-small cell lung cancer, Oksana et al. 27 found that CXCL2 was highly expressed in non-small cell lung cancer tissues compared with corresponding normal tissues. In liver cancer, however, CXCL2 again plays a very different role. Lin et al. 28-30 found through bioinformatics analysis that the expression of CXCL2 in liver cancer tissues was lower than that in normal tissues, and the high expression of CXCL2 indicated that it was beneficial to the prognosis of liver cancer patients. Similarly, CXCL2 expression was low in breast cancer cells. Notably, in previous experiments, knockdown of SHCBP1 was found to significantly increase CXCL2 expression in MCF-7 cells. Therefore, what is the role of CXCL2 in breast cancer cells? Is CXCL2 involved in the regulation of breast cancer cell proliferation by SHCBP1?

Recently, in a study of skin cancer, Huang et al. 31 found that overexpression of SHCBP1 was able to significantly reduce the anti-inflammatory effect of Oroxylin A and speculated that SHCBP1 may be involved in the regulation of inflammatory responses during skin carcinogenesis. Thus, is the involvement of SHCBP1 in the regulation of breast cancer cell proliferation closely related to the occurrence of inflammation? Therefore, we investigated the biological function of CXCL2 and its interaction with SHCBP1 in MCF-7 cells. It is clear that SHCBP1 is involved in the regulation of the occurrence and development of breast cancer by inhibiting the autoinflammatory response of breast cancer cells, which provides an experimental basis for the early diagnosis and treatment of breast cancer.

## Materials and Methods

### Cell culture

The human normal mammary epithelial cell line MDA-KB2 and the breast cancer cell lines MDA-MB-231, MCF-7, and HEK293T, which were obtained from Wuhan Procell Life Science Technology Co., Ltd., were authenticated by STR profiling and regularly tested for mycoplasma contamination. All tumor specimens and corresponding adjacent tissues were from the Department of Pathology of Hanzhong 3201 Hospital. All clinical samples and relevant data collection were granted by the Clinical Research Ethics Committee of Outdo Biotech.

### Flow cytometry

When the cell density reached 80%, the cells were digested with trypsin and centrifuged at 1,000 x g for 3 min at room temperature. Cells were fixed with 70% ethanol overnight at 4°C and subsequently dyed with RNASE and iodide for 30 min at 37°C in the dark. The cell suspension used a liquid cytometer for data analysis, detected the DNA content of each cycle of cells, and determined the proportion of cells in the G0/G1, S, and G2/M phases.

### Cell counting Kit-8 (CCK-8) cell viability assay

When the cell density reached 80%, the cells were seeded in 96-well plates, and each group was set up with 5 compound wells. The cells were cultured for 12 h, 24 h, 48 h, 72 h, and 96 h, and cell activity was detected with a cell counting kit (CCK-8) at 12 h, 24 h, 48 h, 72 h, and 96 h.

### EDU was incorporated to detect cell proliferative activity

When the cell density reached 70%, 1 mL of EDU medium was incubated for 4 h at 37 °C. The cells were fixed with 4% paraformaldehyde (500 μL) at room temperature for 15 min. Then, 500 μL of 0.3% Triton X-100 was added and incubated at room temperature for 15 min. Then, 250 μL of Click reaction solution was added, and the reaction was carried out at room temperature for 30 min away from light. Finally, DAPI staining solution was added in room temperature darkness for nuclear staining for 15 min. EdU-positive staining and nucleus-positive staining were observed under an inverted fluorescence microscope, and the EdU-positive cell rate was calculated.

### Antibodies and Western Blotting

Antibodies directed against the following proteins were used: SHCBP1 (rabbit, 1:1000) and beta-actin (mouse, 1:5000). Ki67 (rabbit, 1:250), p21 (rabbit, 1:1000), p27 (rabbit, 1:2000), cyclin D1 (rabbit, 1:1000), cyclin E1 (rabbit, 1:1000), p-AKT (mouse, 1:2000), and AKT (mouse, 1:5000) were all purchased from Proteintech. Both ERK1/2 (rabbit, 1:1000) and p-ERK1/2 (rabbit, 1:2000) were purchased from Cell Signaling Technology. Primary antibodies were detected by HRP-conjugated secondary antibodies to rabbit and mouse immunoglobulins. Immunoreactivity was determined with the ECL Prime Western Blotting Detection System (GE Healthcare).

### Cell clonogenesis

When the cell density in the petri dish reached 80%. The cells were seeded in 6-well plates at a density of 500 cells per well, and 3 multiple wells were set in each group until the colonies were visible. Crystal violet was dyed, cleaned once with PBS, incubated in cold methanol for 5 min, and then incubated in crystal violet solution (0.4% methanol) for 15 min. Plates were subsequently washed twice with double-distilled water, air-dried, and scanned using a Canon scanner.

### Immunofluorescence and immunohistochemistry

The following antibodies were used for immunofluorescence: SHCBP1 (rabbit, 1:1000), Ki67 (rabbit, 1:250), and CY3 (1:300). Laser focusing microscopy (Leica in Germany) was used to obtain immunofluorescence images.

The following antibodies were used for immunohistochemistry: SHCBP1 (rabbit, 1:1000), sliced slices in Sumu essence. Immunohistochemistry samples were observed with laser focusing microscopy (Leica in Germany).

### Quantitative real-time RT‒PCR

The RNA was reverse-transcribed into cDNA according to the instructions of the reverse transcription kit, which was prepared according to the instructions of the qPCR kit. SHCBP1 sequence: 5'-GCGACACTTTTGTGGACTG-3'; GAPDH sequence: 5'-GCACCGTCAAGG-CTGAGAAC-3'; CXCL2 sequence: 5'-GAACATCCAAAGTGTGAAGGTGA-3'; CCL2 sequence: 5'-GAATCACCAGCAGCAAGTGT-3'; CCL20 sequence: 5'-ATGTCAGTGCTGC-TACTCC-3'. The human GAPDH gene was used as the internal reference gene, and each sample had 3 replicate wells.

### Lentivirus packaging

A lentivirus stable interference SHCBP1 expression vector (PLKO.1-shRNA-SHCBP1) and interference-independent sequence vector (PLKO.1-shRNA-NC) were constructed. Lentivirus transfection packaging plasmid psPAX2 and envelope plasmid pMD2. G were stored in our laboratory. The bacterial solution of pCD513B-CXCL2 and pCD513B was extracted by endotoxin-free plasmid to prepare lentivirus packaging, concentration and infection of MCF-7 cells.

### GEPIA database analysis

The online analysis website (http://gepia.cancer-pku.cn) was used to analyze the data from The Cancer Genome Atlas (TCGA) and the genotype-tissue expression (GENOTEX) database. The GEPIA database was used to analyze the mRNA expression level and prognosis of breast cancer patients in 1085 patients with breast cancer and 291 patients with cancer.

## Results

### Expression and localization of SHCBP1 in breast cancer

**Figure 1:**
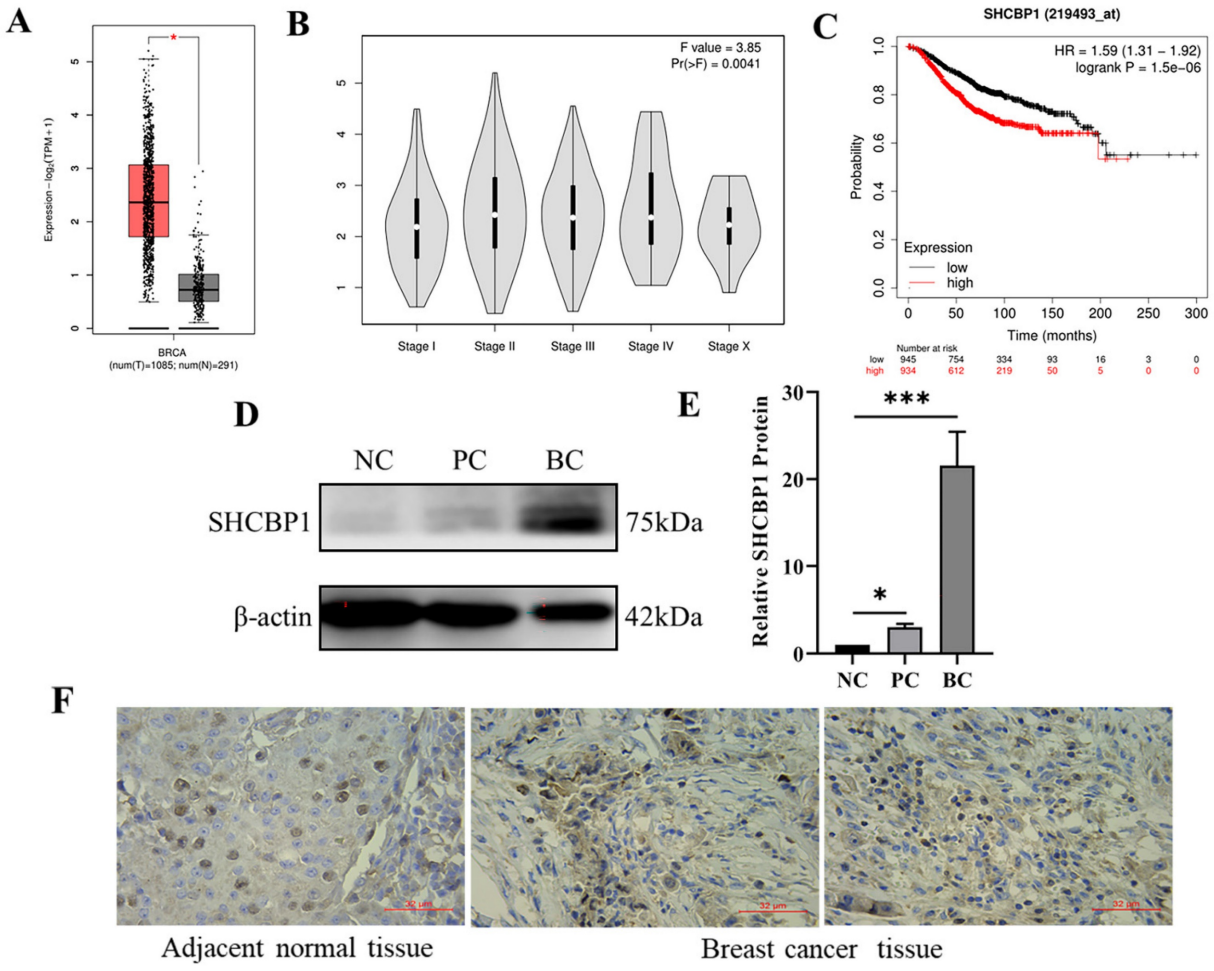
SHCBP1 expression is high in breast cancer tissues. (**A**) The expression of SHCBP1 in breast cancer tissue and normal breast tissue in GEPIA database, *P<0.05. (**B**) Correlation between the abundance of SHCBP1 gene expression and the clinical stage of breast cancer in GEPIA database, **P<0.01. (**C**) Correlation between SHCBP1 gene expression abundance and prognosis of breast cancer patients in TCGA database, **P<0.01. (**D**) The expression of SHCBP1 protein in breast cancer tissue and normal breast tissue was detected by Western blot. NC represents normal breast tissue, PC represents adjacent breast cancer tissue, and BC represents breast cancer tissue. (**E**) Protein gray analysis, *P<0.05, ***P<0.001. (**F**) The localization and expression of SHCBP1 in breast cancer tissue and normal breast tissue were analyzed by immunohistochemical staining.

Previous research has shown that the level of SHCBP1 mRNA is markedly increased in several cancer cell lines and can promote tumorigenesis and development 32. To determine whether and how SHCBP1 affects the proliferation of breast cancer, we analyzed the expression abundance of SHCBP1 in breast cancer tissues and normal breast tissues from the GEPIA database. Notably, the SHCBP1 mRNA expression levels were significantly elevated in breast cancer tissues (P<0.05) (Fig. 1A). Meanwhile, we found that the expression level of SHCBP1 was correlated with the clinical stage of breast cancer, with the highest expression in stage II (P<0.01) (Fig. 1B). Intriguingly, the survival rate of patients was closely related to the expression level of SHCBP1 during the breast cancer survival curve over time (HR=1.59, P=1.5e-06) (Fig. 1C). To further confirm the database results, we detected that the protein expression of SHCBP1 was highest in breast cancer tissues by Western blotting (Fig. 1D, E). Immunohistochemistry showed that SHCBP1 was mainly distributed in the nucleus and exhibited a high expression level in breast cancer tissues (Fig. 1F). In summary, we speculate that SHCBP1 may play an important role in intracellular signal transduction and cell division in breast cancer cells.

**Figure 2:**
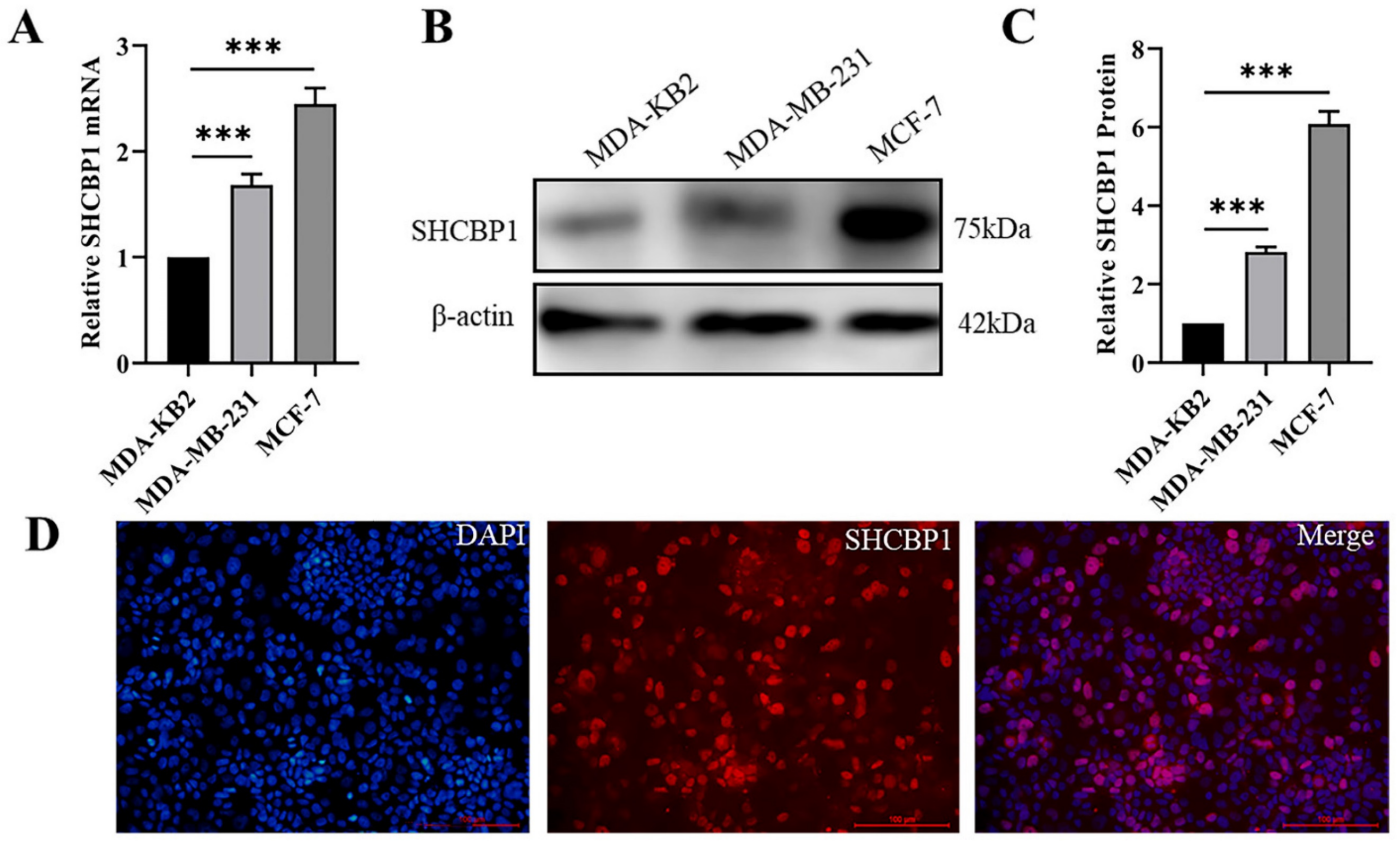
Localization and expression analysis of SHCBP1 in breast cancer cells. (**A**) mRNA expression levels of SHCBP1 in MDA-KB2, MDA-MB-231 and MCF-7 cells were detected by qPCR, ***P<0.001. (**B**) The protein expression of SHCBP1 in MDA-KB2, MDA-MB-231 and MCF-7 cells was determined by Western blot. (**C**) Protein gray scale analysis, ***P<0.001. (**D**) Expression and localization of SHCBP1 in MCF-7 cells.

MCF-7 and MDA-MB-231 are human breast cancer cell lines with low and high metastatic potential, respectively 33. Remarkably, the mRNA and protein levels of SHCBP1 were prominently higher in MDA-MB-231 and MCF-7 cells than in normal breast epithelial cells (MDA-KB2) (Fig. 2A-C). Because the expression of SHCBP1 was the highest in MCF-7 cells, we used MCF-7 cells to analyze the subcellular localization of SHCBP1. Immunofluorescence staining showed that SHCBP1 was highly expressed in the nucleus of MCF-7 cells (Fig. 2D), which was consistent with the localized expression of SHCBP1 in breast cancer tissues. These results indicated that SHCBP1 was highly expressed in breast cancer tissues and breast cancer cells and was mainly located in the nucleus of breast cancer cells.

### Downregulation of SHCBP1 expression delays MCF-7 cell proliferation

**Figure 3:**
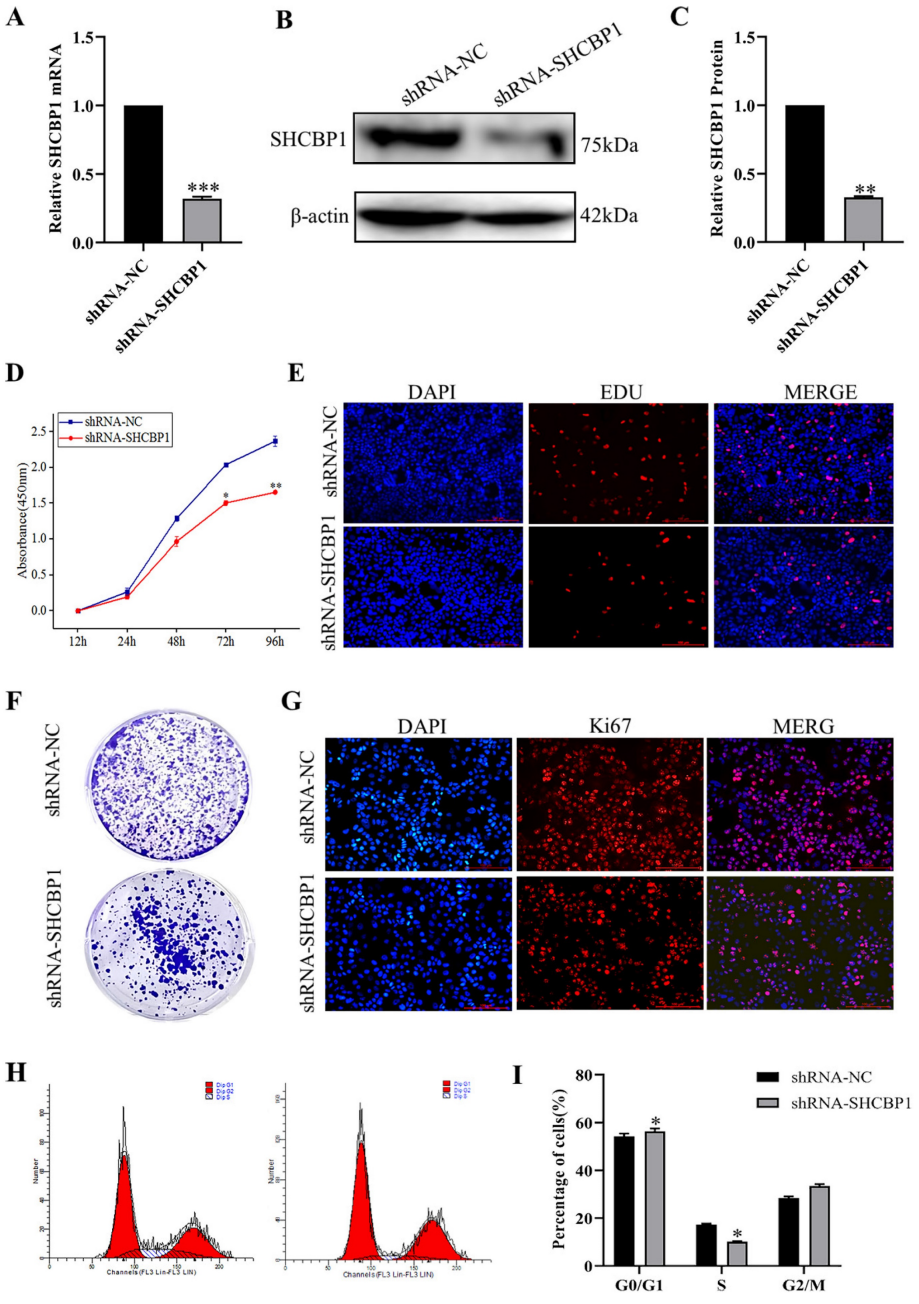
The effect of interfering with SHCBP1 on the proliferation of MCF-7 cells. (**A**) The mRNA expression level of SHCBP1 was detected by qPCR, ***P<0.001. (**B**) The protein expression of SHCBP1 was detected by Western blot. (**C**) Protein gray analysis, **P<0.01. (**D**) Cell proliferation activity was detected by CCK-8, *P<0.05, **P<0.01. (**E**) DNA synthesis is measured by EDU staining, with blue fluorescence representing the nucleus and red fluorescence representing EDU positive cells. (**F**) Cell clonal formation experiment. (**G**) Ki67 protein expression in MCF-7 cells after interference with SHCBP1. Blue fluorescence represents the nucleus and red fluorescence represents positive expression of Ki67 protein. (**H**) The distribution of MCF-7 cell cycle in different phases after SHCBP1 interference. (**I**) Cell distribution was quantified at each stage, *P<0.05.

Based on the above results, SHCBP1 may play an oncogenic role in the breast cancer progression. To further investigate the biological function of SHCBP1 in breast cancer cells, we knocked down SHCBP1 in MCF-7 cell lines, and the efficiency of SHCBP1 knockdown was 68% (P<0.01) (Fig. 3A-C). We found that the decreased SHCBP1 level downregulated the rate of cell proliferation at 72 h by CCK8 assay (Fig. 3D). EDU is a thymidine analog that can replace deoxythymidine and insert into DNA molecules during cell proliferation, thus allowing the effective measurement of the percentage of cells in S phase 34. EdU staining demonstrated that SHCBP1 deficiency reduced the percentage of positive cells in S phase (Fig. 3E, Supplementary Figure S1A). Simultaneously, cell colony formation experiments further confirmed that loss of SHCBP1 expression reduced the clonogenicity of MCF-7 cells (Fig. 3F, Supplementary Figure S1B). Ki67 is a cellular marker of proliferation commonly used as a biomarker for estimating cancer cell outcome 35. The results showed that the number of Ki67-positive cells was reduced by 22.5% after knockdown of SHCBP1 expression in MCF-7 cells (Fig. 3G, Supplementary Figure S1C). These results indicated that SHCBP1 ablation restrained MCF-7 cell proliferation.

To assess which phase of the cell cycle might be influenced by SHCBP1, cell cycle progression was analyzed by flow cytometry (FACS). FACS analysis indicated a substantial increase in cells at G1 phase and a visible decrease in cells at S phase upon SHCBP1 silencing compared to control cells (Fig. 3H, I), which implied that SHCBP1 deficiency could induce G1/S cell cycle arrest and retard MCF-7 cell proliferation. In conclusion, these results indicated that SHCBP1 was required for MCF-7 cell proliferation and that SHCBP1 may act as a G1/S transition regulator.

### SHCBP1 regulated CXCL2 expression in MCF-7 cells

**Figure 4:**
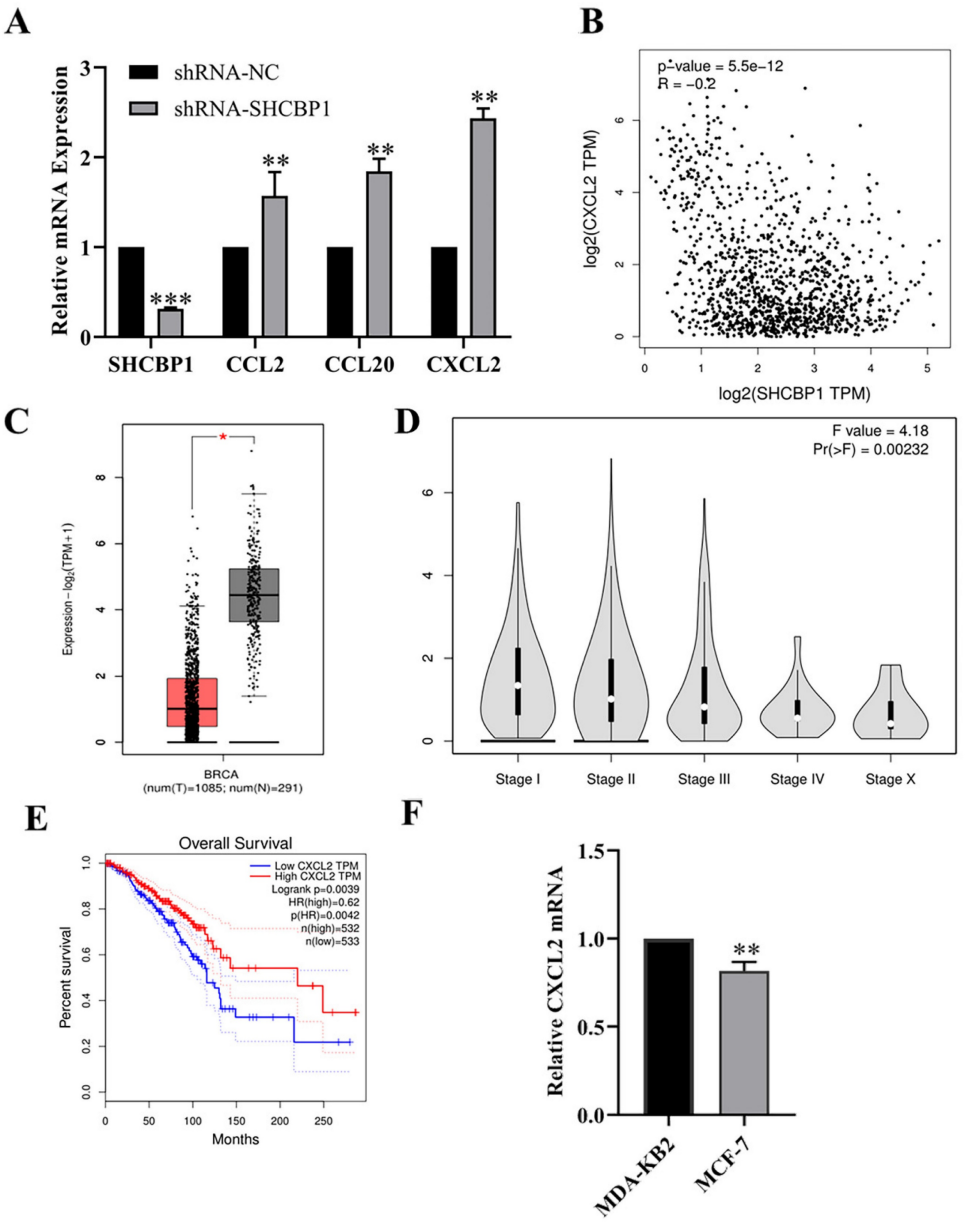
Expression of CXCL2 in breast cancer. (**A**) The mRNA expression levels of chemokines were detected by qPCR, **P<0.01, ***P<0.01. (**B**) The correlation between CXCL2 and SHCBP1 expression was analyzed in GEPIA database, **P<0.01. (**C**) Difference expression of CXCL2 in breast cancer tissue and normal breast tissue in GEPIA database, *P<0.05. (**D**) Relationship between CXCL2 expression in GEPIA database and clinical stage, **P<0.01. (**E**) Relationship between CXCL2 expression level and prognosis of breast cancer patients in GEPIA database, *P<0.05. (**F**) mRNA expression levels of CXCL2 in MDA-KB2 and MCF-7 cells were detected by qPCR, **P<0.01.

Huang et al. 31 demonstrated that SHCBP1 may be involved in the regulation of inflammation in the process of skin carcinogenesis. Hence, in breast cancer, is the regulation of breast cancer cell proliferation by SHCBP1 mediated by inflammatory cytokines? Notably, after knocking down SHCBP1 expression in MCF-7 cells, the mRNA expression of the chemokine family members CCL2, CCL20 and CXCL2 was upregulated, and the expression of CXCL2 was upregulated by 2.43-fold, suggesting that SHCBP1 had the most significant effect on regulating the expression of CXCL2 (P<0.01) (Fig. 4A).

Next, we explored the correlation between SHCBP1 and CXCL2 expression in breast cancer using the GEPIA database (Fig. 4B). As expected, the SHCBP1 expression is negatively correlated with the expression of CXCL2 in breast cancer cell (P<0.01), suggesting that CXCL2 may play an important role in the proliferation of MCF-7 cells regulated by SHCBP1.

**Figure 5:**
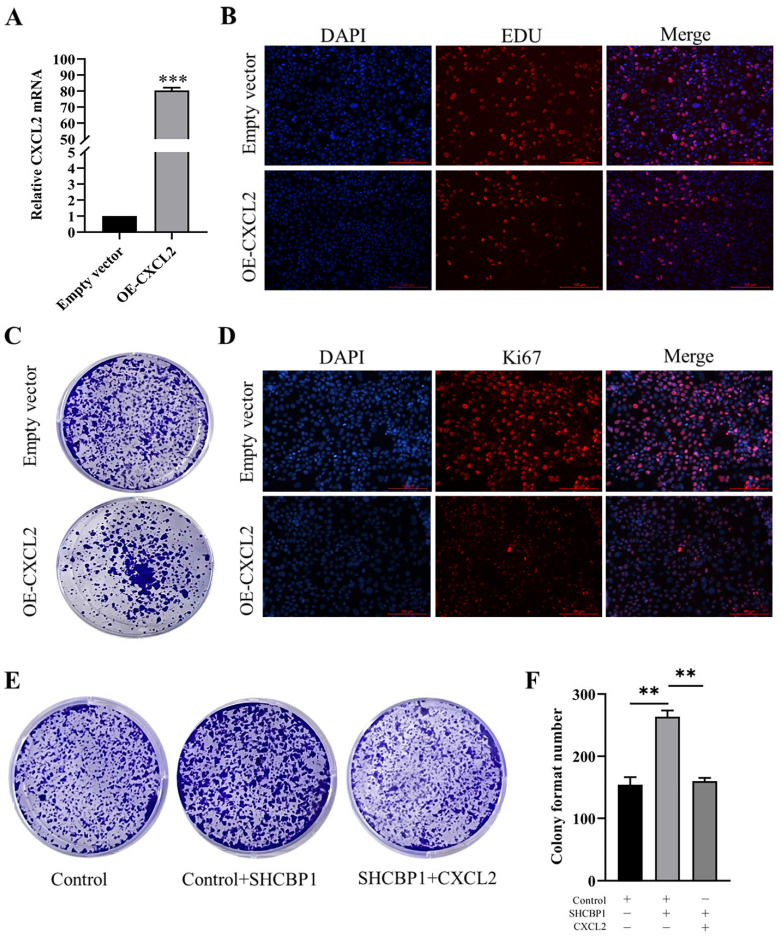
The effect of overexpression of CXCL2 on the proliferation of MCF-7 cells. (**A**) mRNA level of CXCL2 was detected by qPCR, ***P<0.001. (**B**) EDU staining was used to detect DNA synthesis. (**C**) Cell clonal formation experiment. (**D**) Expression of Ki67 protein in MCF-7 cells after CXCL2 overexpression. (**E**) Cell clonal formation experiment. (**F**) The results of cell cloning formation experiment were quantified, **P<0.01.

To illustrate the functional significance of CXCL2 during MCF-7 cell proliferation, accordingly, we found that CXCL2 was significantly downregulated in breast cancer tissues by the GEPIA database (P<0.05) (Fig. 4C). In parallel, the expression of CXCL2 was closely related to the stage of breast cancer, with deficiency in stage X (Fig. 4D). In contrast to SHCBP1, breast cancer patients with low CXCL2 expression had poor overall survival (Fig. 4E). The results of qPCR in breast cancer cells were consistent with the database (Fig. 4F). These data indicated that CXCL2 is expressed at a low level in breast cancer tissues and cells. Next, we focused on whether CXCL2 affects MCF-7 cell proliferation. We overexpressed CXCL2 in MCF-7 cells by lentivirus (Supplementary Figure S2A), and CXCL2 mRNA levels were analyzed by qPCR (Fig. 5A). Cell proliferation was measured by EdU, which showed a significant decrease in cell growth after CXCL2 overexpression (Fig. 5B, Supplementary Figure S2B). The results of the colony formation assay also showed that the proliferation ability of MCF-7 cells was inhibited (Fig. 5C, Supplementary Figure S2C). Ki67 staining was also performed, which showed that the number of Ki67-positive cells was reduced by 27.1% (Fig. 5D, Supplementary Figure S2D). Taken together, these results indicate that CXCL2 inhibits MCF-7 cell proliferation and may act as a tumor suppressor gene.

To further investigate whether SHCBP1 promote cell proliferation by inhibiting CXCL2 expression, three controlled trials were set up in this study. Compared with the control group (154±15), the colony formation ability in SHCBP1-sufficient MCF-7 cells exhibited obvious enhancement (264±20) (P<0.01). However, CXCL2 upregulation reversed the increased proliferation of MCF-7 cells induced by SHCBP1 overexpression (P<0.01) (Fig. 5E, F), which robustly verified the critical involvement of CXCL2 in the regulation of MCF-7 cell proliferation by SHCBP1.

### SHCBP1 promotes the proliferation of MCF-7 cells by inhibiting the expression of CXCL2 and activating the ERK1/2 signaling pathway

**Figure 6:**
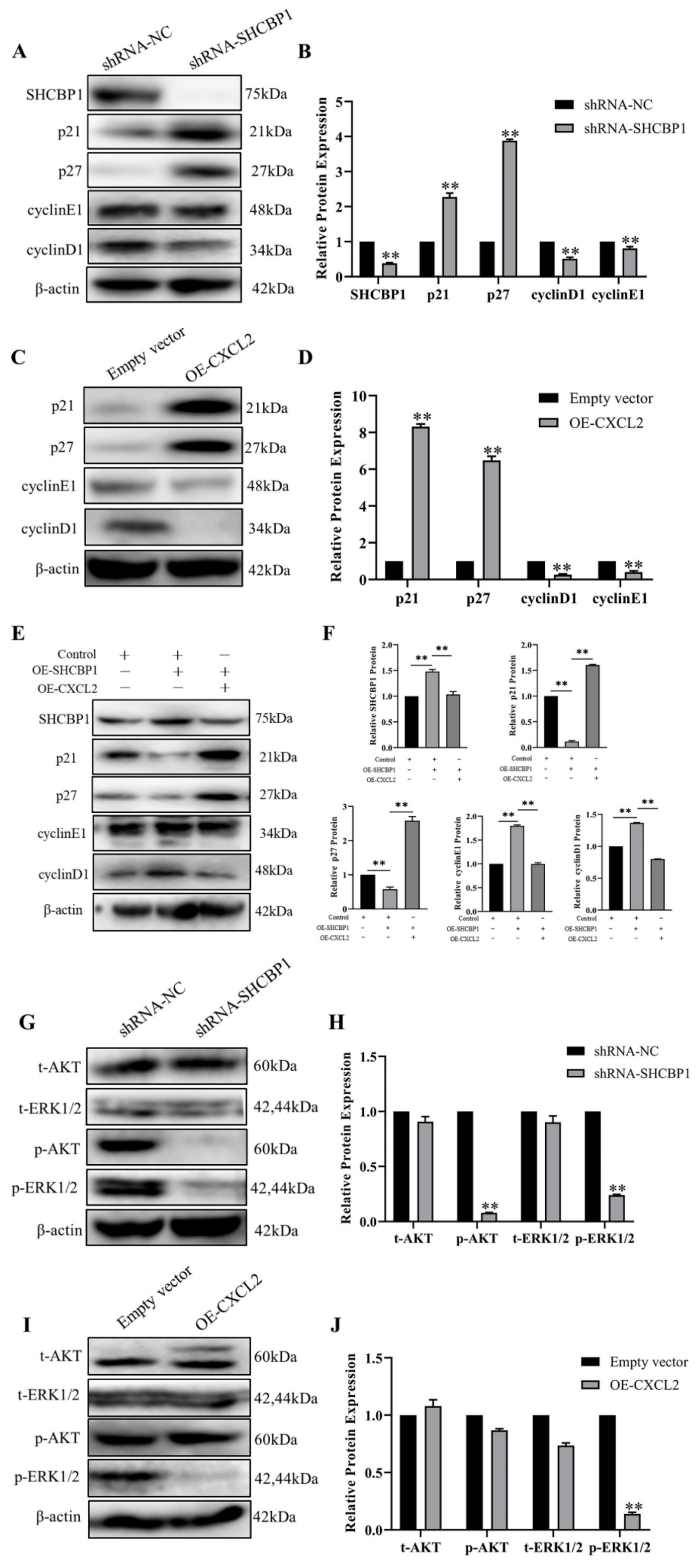
Effect of CXCL2 and SHCBP1 expression on MCF-7 cell cycle-related proteins. (**A**) Western blot was used to detect the expression of cell cycle-related protein after interference with SHCBP1. (**B**) Protein gray analysis, **P<0.01. (**C**) Western blot was used to detect the expression of cycle-related proteins after overexpression of CXCL2. (**D**) Protein gray analysis, **P<0.01. (**E**) Western blot was used to detect the expression of cycle-related proteins. (**F**) Protein gray analysis, **P<0.01. (**G**) Western blot was used to detect the expression levels of AKT and ERK1/2 signaling pathways after interference with SHCBP1. (**H**) Protein gray analysis, **P<0.01. (**I**) The expression levels of AKT and ERK1/2 signaling pathways after CXCL2 overexpression were detected by Western blot. (**J**) Protein gray analysis, **P<0.01.

Since we showed that SHCBP1 promoted cell proliferation by enhancing the G1/S transition during the cell cycle, we next focused on investigating the molecular mechanism by which SHCBP1 enhanced the G1/S phase of the cell cycle. It is well documented that p21 and p27 are both cell cycle kinase inhibitors, and enhanced expression levels of the two proteins can retard cell proliferation 36. We next examined the functional significance of p21 and p27 in this process. Notably, the expression of p21 and p27 was more dramatically increased at the protein level in MCF-7 cells after SHCBP1 upregulation (Figure 6A, B), suggesting that the expression levels of p21 and p27 may be regulated by SHCBP1 during cell proliferation. In addition, low expression of SHCBP1 decreased cyclin E1 and cyclin D1 protein levels in MCF-7 cells (Figure 6A, B). Meanwhile, there were obvious increases in the protein levels of p21 and p27 when abundant CXCL2 was expressed on MCF-7 cells, and these increases were accompanied by significant reductions in cyclin D1 and cyclin E1 (Figure 6C, D). These results strongly suggested that SHCBP1 and CXCL2 restrained cell cycle progression in MCF-7 cells by targeting p21, p27 cyclin D1 and cyclin E1 through alterations in their expression.

To further clarify the relationship between SHCBP1 and CXCL2, the two genes were co-overexpressed in MCF-7 cells. Remarkably, compared with overexpression of SHCBP1 alone, the expression levels of p21 and p27 were significantly increased after co-overexpression of SHCBP1 and CXCL2. Moreover, the protein expression of cyclin D1 and cyclin E1 showed a decreasing trend (Figure 6E, F). Therefore, we speculated that the effect of SHCBP1 on MCF-7 cell proliferation was achieved under low levels of CXCL2 expression. Combined with the previous results of SHCBP1 inhibiting the expression of CXCL2, along with CXCL2 inhibiting the proliferation of MCF-7 cells, it is suggested that SHCBP1 promotes the proliferation of MCF-7 cells by reducing the expression of CXCL2 and regulates the expression of specific genes related to the cell cycle in MCF-7 cells.

It is well known that the AKT and ERK signaling pathways play crucial roles in regulating physiological and pathological processes and are important signaling pathways that affect biological processes. Activated AKT and ERK signaling pathways activate or inhibit the expression of their downstream target proteins p21, p27 and cell cyclin-related proteins through phosphorylation, thereby regulating cell proliferation, differentiation, apoptosis and other biological processes 37. To our knowledge, SHCBP1 is involved in activating the MAPK/ERK and PI3K/AKT signaling pathways. To further clarify the signaling pathway by which SHCBP1 regulates MCF-7 cell proliferation, in the present study, the expression levels of AKT, p-AKT, ERK, and p-ERK1/2 were detected by western blotting. As shown in Figure 6G, depletion of SHCBP1 led to a significant attenuation in p-AKT and p-ERK1/2 in MCF-7 cells.

However, only the expression of p-ERK1/2 was much lower in the OE-CXCL2 group than in the empty vector group (Figure 6I, J), and there was no significant change in the expression of AKT, ERK and p-AKT. The results showed that CXCL2 inhibited the proliferation of MCF-7 cells by the ERK1/2 signaling pathway. Finally, these findings indicated that SHCBP1 inhibited the expression of CXCL2, activated the ERK1/2 signaling pathway and regulated the expression of cell cycle-related proteins to achieve its function in MCF-7 cells.

## Discussion

SHCBP1 activity has been widely linked to several human diseases, and its expression is significantly increased in a variety of solid tumors 38. The abnormally high expression of SHCBP1 can be used for the clinical diagnosis and prognosis of cancer patients and may play an oncogenic role in the process of tumorigenesis 39, 40. Mei et al. 41 found in a study of breast cancer that the expression of SHCBP1 in breast cancer tissues was higher than 64% in normal tissues. Similar results were obtained in the present study, with abnormally high expression of SHCBP1 in breast cancer tissues and cells, and its upregulation level was correlated with the clinicopathologic staging of breast cancer and the survival rate of patients. Specifically, high SHCBP1 activity was associated with worse survival rates. Remarkably, inhibition of cell proliferation and colony formation was observed in MCF-7 cells with SHCBP1 interference. Flow cytometry showed that the cell cycle was arrested in the G1/S phase, suggesting that SHCBP1 is essential for the proliferation of MCF-7 cells and may act as a G1/S transition regulator.

Recently, it was shown that tumors caused by chronic inflammation or persistent infection account for approximately 20% of the risk factors 42, 43. As inflammatory cytokines in the process of immune regulation can be secreted by a variety of cells, such as tumor cells, and induce the directional migration of immune cells, CXCL2 has been reported to play a complex and variable role in tumor progression, even in different cell lines within the same tumor 24, 44. Studies have shown that CXCL2 can enhance the proliferation and migration of SMMC7721 hepatoma cells 45.

Nonetheless, in two HCC cell lines, MHCC97H and HCCLM3, overexpression of CXCL2 was found to inhibit cell proliferation and promote cell apoptosis, and consistent results were achieved *in vivo* 29. The above research results show that the expression of the CXCL2 gene is tissue- and signal specific. Therefore, what is the role of CXCL2 in breast cancer progression? Found in our data, CXCL2 is expressed at low levels in breast cancer cells and is positively correlated with the survival rate of patients. Deficiency of SHCBP1 could upregulate the mRNA expression levels of chemokines (CCL2, CCL20 and CXCL2). In particular, the upregulation of CXCL2 alone was sufficient to inhibit the proliferation activity and colony formation efficiency of MCF-7 cells, further indicating the critical role of CXCL2 in breast cancer. Furthermore, in the cells co-overexpressing SHCBP1 and CXCL2, we observed a significant reduction in MCF-7 proliferation compared with SHCBP1 overexpression alone, which strongly supports our hypothesis that SHCBP1 promotes the proliferation of MCF-7 cells by regulating the occurrence of inflammation.

Combined with the previous results that SHCBP1 inhibited the expression of CXCL2 in MCF-7 cells and that CXCL2 inhibited the proliferation of MCF-7 cells, we further investigated whether SHCBP1 affects CXCL2 function by inhibiting its expression. Zhang et al. 46 found that treatment with RvD1 attenuated LPS-induced neutrophil infiltration via the downregulation of CXCL2 expression on resident alveolar macrophages. In addition, lnccxcl2 limits lung inflammation by suppressing Cxcl2 expression 47. Our analysis also revealed that the expression of cell cycle-related proteins was examined in the presence of high expression levels of SHCBP1 and CXCL2 simultaneously. As expected, coexpression of CXCL2 and SHCBP1 compared with only SHCBP1-increased cells, the expression levels of cyclinD1 and cyclinE1 proteins were decreased, while p21 and p27 proteins were increased. These results indicate that SHCBP1 can promote MCF-7 cell proliferation when CXCL2 is expressed at low levels, which validates our hypothesis that SHCBP1 promotes the development of cancer by inhibiting the inflammation of tumor cells.

It has been reported that PI3K/AKT and ERK protein kinases can participate in the regulation of cell proliferation, apoptosis and differentiation during the occurrence and development of tumors 48, 49. The mechanism involves the regulation of cyclin D by the ERK1/2 signaling pathway and the expression of cell cycle-related factors such as p16 and CDK 49. In addition, activated AKT and ERK1/2 signaling pathways activate or inhibit the expression of downstream target proteins p21 and p27 and cell cycle-related proteins through phosphorylation 50, 51. Our findings demonstrate that interference with SHCBP1 expression in MCF-7 cells inhibited ERK1/2 and AKT phosphorylated protein activity, suggesting that SHCBP1 inhibits the proliferation of MCF-7 cells by regulating the expression of cell cycle-related proteins through the ERK1/2 and AKT signaling pathways. It's worth noting that overexpression of CXCL2 apparently regulated the phosphorylation of ERK1/2 protein expression, which further confirmed that SHCBP1 can suppress the inflammatory response and then regulate MCF-7 cell proliferation through the ERK1/2 signaling pathway. Further studies are needed to elucidate the mechanism by which SHCBP1 regulates AKT signaling.

## Supplementary Material

Supplementary figures.
