# Supplementary material for: SHCBP1 Promotes the Proliferation of Breast Cancer Cells by Inhibiting CXCL2
Source: J Cancer. 2023 Oct 16;14(18):3444–56. doi: 10.7150/jca.88072 (PMC10647193; doi:10.7150/jca.88072)
Supplement: Supplementary file 1 — Supplementary figures. [file jcav14p3444s1.pdf]

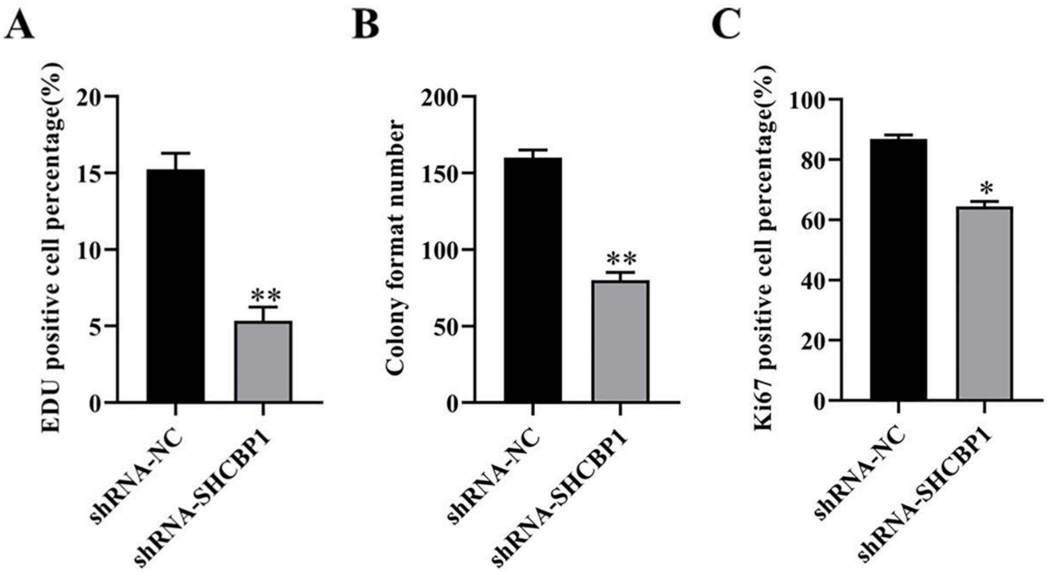

Supplementary Figure S1 (**A**) EDU positive cell rate, **P<0.01. (**B**) The results of cell cloning formation experiment were quantified, **P<0.01. (**C**) The positive rate of Ki67 protein was *P<0.05.

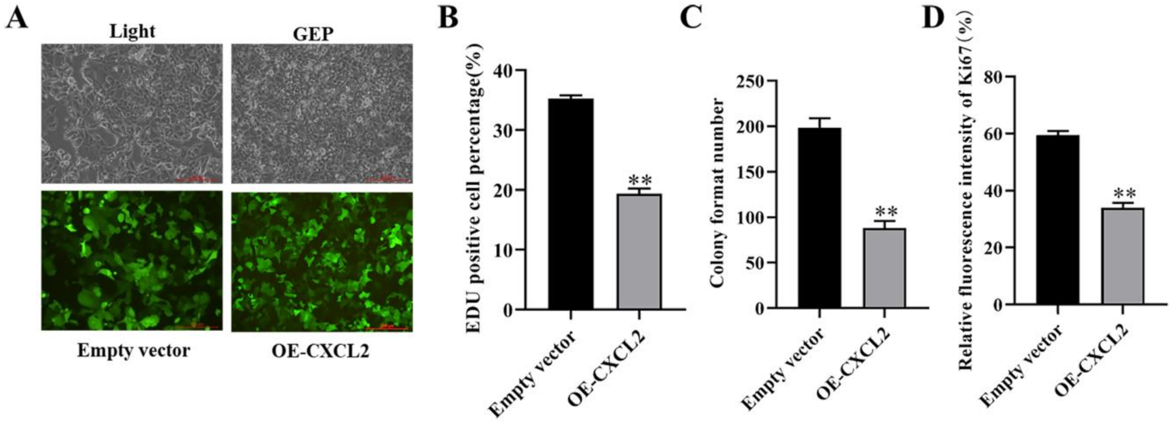

Supplementary Figure S2 (**A**) Fluorescence detection of MCF-7 cells infected with lentivirus. (**B**) EDU positive cell rate, **P<0.01. (**C**) The results of cell cloning formation experiment were quantified, **P<0.01. (**D**)The positive rate of Ki67 protein was *P<0.05.
